# Telecoaching as a new training method for elderly people: a systematic review

**DOI:** 10.1007/s40520-023-02648-9

**Published:** 2024-02-02

**Authors:** Ignazio Leale, Flavia Figlioli, Valerio Giustino, Jessica Brusa, Matteo Barcellona, Valerio Nocera, Alberto Canzone, Antonino Patti, Giuseppe Messina, Mario Barbagallo, Antonio Palma, Ligia J. Dominguez, Giuseppe Battaglia

**Affiliations:** 1 Sport and Exercise Sciences Research Unit, Department of Psychology, Educational Science and Human Movement, University of Palermo, Via Giovanni Pascoli, 6, 90144 Palermo, Italy; 2 PhD Program in Health Promotion and Cognitive Sciences, University of Palermo, Via Giovanni Pascoli, 6, 90144 Palermo, Italy; 3 Department of Human Sciences and Promotion of the Quality of Life, San Raffaele University, Via di Val Cannuta, 247, 00166 Rome, Italy; 4 PLab Research Institute, Palermo, Italy; 5 Geriatric Unit, Department of Internal Medicine and Geriatrics, University of Palermo, Via del Vespro, 129, 90127 Palermo, Italy; 6 Regional Sports School of Italian National Olympic Committee (CONI) Sicilia, Palermo, Italy; 7 School of Medicine, University Kore, Enna, Italy

**Keywords:** Physical activity, Exercise, Sustainable exercise, Risk of falls, Falls prevention, Older people

## Abstract

**Background:**

The numerous restrictive measures implemented during the recent COVID-19 pandemic have reduced the levels of physical activity (PA) carried out by elderly people and telecoaching (TC) could be a training method to maintain the recommended levels of PA. In fact, TC uses information and digital communications technologies, such as computers and mobile devices, to access training services remotely. Thus, this study aimed to systematically review the scientific literature to verify the application, efficacy, and safety of TC training programs.

**Methods:**

PubMed, Scopus, and Web of Sciences databases were used for this review, and randomized controlled trials analyzing TC training programs for elderly people were included. Only articles written in English and published in the last decade were considered.

**Results:**

3 articles were included in the qualitative synthesis including 194 elderly people. The sample size ranged from 12 to 117 and the TC training program from 8 to 12 weeks. The TC training programs were applied to elderly people with metabolic diseases and respiratory diseases. TC training program was effective in elderly people with metabolic diseases while the benefits for respiratory diseases have yet to be clarified.

**Conclusion:**

TC seems to be a safe, effective, and injury-free training method, despite its limited application in elderly population. Future studies should better investigate this training method in elderly people in order to evaluate the effectiveness in a wider range of diseases.

## Introduction

The world aging process is indisputable, 125 million people are 80 years of age or older [1], altering the social, economic, and health systems of most countries [2]. Population aging is a problem for the present and especially for the future, and the United Nations estimates that the elderly population will reach 2 billion by 2050. This aging process results from the increase in life expectancy, the decrease in birth rates, and the advances in medical care and technology [1]. The European Commission has recently expressed the need to increase scientific research to ensure healthy aging [1]. Indeed, after the age of 40, it is possible to detect a first involution of the physiological systems, involution that develop at a peak near 65 years of age [3, 4]. Aging is a process associated with structural and functional changes in both the physical and mental domains [5]. The mental component is altered due to neurological mechanisms related to aging [6], such as vestibulocochlear degeneration [7], the reduction of cognitive functions [8], and the development of dementia and depression [8], while the physical component is associated with a decreased muscle strength, muscle flexibility, and body balance [7, 9, 10]. All these impairments, associated with a sedentary lifestyle, accelerate the decline of bodily functions [11] and increase the risk of falls [12, 13]. Consequently, to ensure a healthy aging, it is important to develop appropriate health habits such as sleep management, stress management, balanced nutrition, and physical activity (PA) practice [14].

PA has been shown to be effective in encouraging a healthy aging [15]. For example, a regular outdoor walk seems to generate benefits on body balance ability in elderly people and consequently prevent the risk of falls [16]. The benefits of PA in elderly have also been demonstrated in sarcopenia [13] and osteoporosis diseases [17], as well as to improve physical performance such as the range of motion (ROM) of joints. For example, a flexibility training program has been shown to improve spinal ROM in a population of older women [18].

In consideration of the recent COVID-19 pandemic and the relative restrictive measures, it would be useful to increase the duration of the weekly time of PA practice in elderly people [19, 20]. Indeed, the recent COVID-19 pandemic, due to the numerous restrictive measures implemented, has reduced the levels of PA practiced by this population [21, 22]. As a consequence, different strategies have been developed and, among these, telecoaching (TC) seems to result as an effective training setting for maintaining the recommended levels of PA [23, 24]. TC provides the use of information technologies and digital communications, such as computers and mobile devices, to access training services remotely [25]. It is a useful training method for all patients with travel difficulties, who live in different cities, or who prefer to train in a familiar environment such as their own home [26].

Therefore, this systematic review aimed to analyze the application, efficacy, and safety of TC training programs in elderly people.

## Methods

### Search strategy

For conducting this systematic review the PRISMA guidelines were adopted [27]. The studies were searched in electronic databases such as PubMed, Scopus, and Web of Science. To find studies, the following keywords were used in different combinations: telecoaching, exercise, elderly, training protocol, and training program.

All the articles found were transferred into Endnote software (vers. X9 for Windows 11, © Thomson Reuters).

### Eligibility Criteria

All studies that have the following criteria were included in this systematic review: (1) studies with TC as training program for elderly people; (2) original research with full text written in English language; (3) studies published in the last decade; (4) study designs other than reviews, meta-analysis, letter to editors, and theses. No gender differences between males and females were considered for this study.

### Study selection

The search of the studies was performed by one author of the research group (I.L.). After removing duplicates, two authors (F.F. and V.G.) independently analyzed the titles and abstracts of all studies. Other two authors (J.B. and M.B.) later analyzed the full text for study inclusion according to the inclusion and exclusion criteria. In case of disagreement of the latter two authors, a negotiation process was carried out and, if necessary, a third author (V.N.) was consulted.

A Microsoft Excel spreadsheet (Microsoft Corp, Redmond, Washington) was used to record the following information related to the included studies: year of publication, sample age, gender, aim of the study, and TC program.

## Results

### Study Identification

**Fig. 1:**
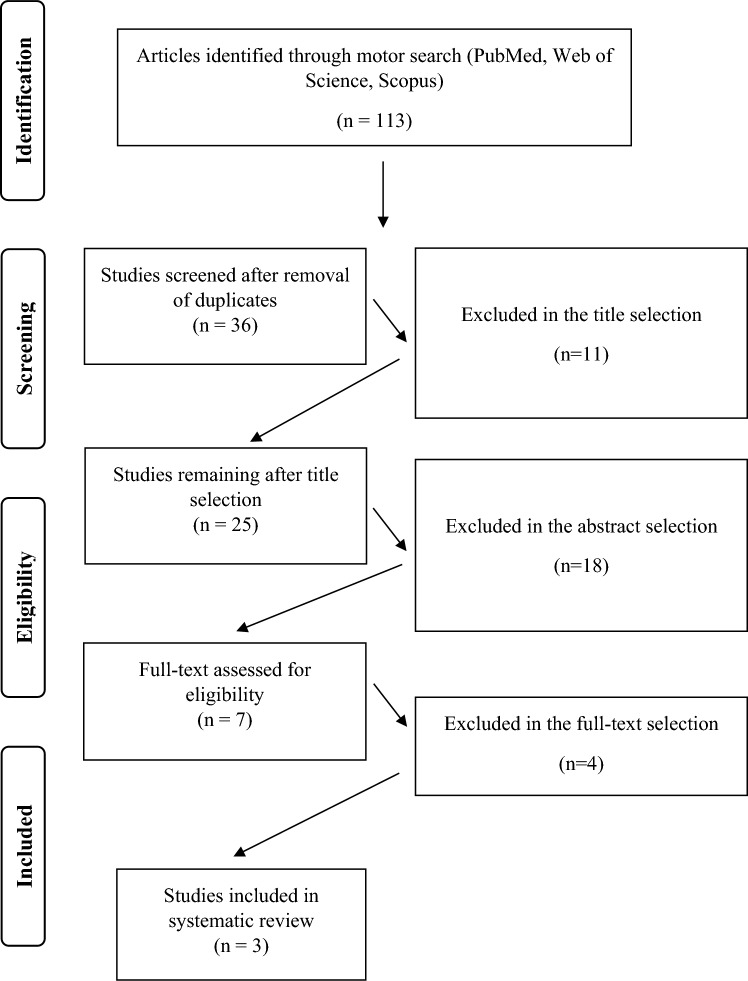
Flow Diagram representing the entire selection process

From the search conducted on these databases, a total of 113 studies analyzing the use of TC in elderly were found. 77 were doubled and 33 were eliminated because they did not include elderly subjects, or they analyzed a different topic. Only 3 articles were finally included. In detail, 2 studies aimed to evaluate the effects of TC in elderly suffering from respiratory diseases, while 1 study aimed to evaluate the effects of TC in elderly suffering from metabolic disease. The PRISMA flowchart shows the entire selection process (Fig. 1).

A total of 194 elderly participants were included and the sample size varied from 12 to 117 while the TC training program lasted from 8 to 12 weeks. Detailed information is provided in Table 1.

**Table 1:** Characteristics of the studies included

1st author, year	Participants [f] [m]	Age (SD)	Weeks	Aim	Telecoaching
Cameron-Tucker et al. (2016) [28]	65 [36] [29]	68 (8.6)	8	To evaluate the effectiveness of telecoaching in patients with COPD	Telecoaching supported participants’ home-based free walking through pedometers and two calls weekly
Hume et al. (2022) [29]	12 [4] [8]	57 (9.0)	12	To evaluate the effectiveness of a telecoaching program on LTx patients	Telecoaching supported physical activity programs through a smartphone app, pedometers, and calls weekly
Storch et al. (2019) [30]	117 [23] [94]	59 (6.3)	12	To evaluate the effects of telecoaching in patients with T2DM	Telecoaching supported a lifestyle intervention through an individual coaching approach

*F* female, *M* male, *SD* standard deviation, *COPD* chronic obstructive pulmonary disease, *LTx* lung transplantation, *TD2M* type 2 diabetes mellitus

Table 2 shows details of the training program and the results found in the articles included.

**Table 2:** Description of intervention, training program, and results of the studies included

1st author, year	Intervention	Training program	Results
Cameron-Tucker et al. (2016) [28]	Telecoaching (35) vs. usual care (30) + physical activity	Patients were asked to walk at a moderate intensity (i.e., to breathe more heavily but not to “huff and puff”) to accumulate 30 min daily, preferably all days of the week	There were no significant changes between time points or between groups
Hume et al. (2022) [29]	Telecoaching (7) vs. usual care (5)	Patients were asked to wear the pedometer during waking hours and interact with the smartphone application every day by reviewing and completing the automated application tasks (free walking + home exercise)	Telecoaching seems to be a feasible, safe, and well-accepted intervention in LTx
Storch et al. (2019) [30]	Telecoaching (60) vs. usual care (55)	Patients were asked to wear a pedometer and walk. Periodically through a telephone meeting, the coach and participant have decided the goals	Patients with TD2M can benefit from this new type of intervention. Telecoaching has proven useful in disease management

*LTx* lung transplantation, *TD2M* type 2 diabetes mellitus

### Respiratory diseases

The first study included in this review was conducted by Cameron-Tucker and colleagues [28] who applied the TC method to patients with chronic obstructive pulmonary disease (COPD) which is a chronic inflammatory disease with non-reversible obstruction of airways [30]. The researchers investigated telephone health mentoring aimed at home-based free walking (TC or experimental group) compared to the usual waiting time (usual care or control group) followed by a training program for each group. Participants were randomized between the two groups (i.e., TC and usual care). TC provided by trained nurse healthcare supported participants’ home-based free walking for 8 weeks, to accumulate 30 min of training per day, preferably every day of the week. Data collection was performed at the baseline (T1), after the TC program (T2), and at the end of the training program of both groups (T3). The primary outcome was a change in physical capacity measured by a 6-min walk test, resulting in highly indicative disease status in patients suffering from respiratory diseases [31]. The results demonstrated no significant changes between time points in the TC group. It follows that in patients with COPD, telephone mentoring for home-based walking has not demonstrated any benefit on exercise capacity.

The second study included in this review was conducted by Hume and colleagues [29]. The researchers evaluated the feasibility and acceptability of a TC training program for lung transplant recipients (LTx). The program included 12 weeks of TC training program. In detail, patients were recruited and randomized into two groups (i.e., TC and usual care). TC involved the use of a pedometer and a smartphone app, with the latter the researchers established periodic PA goals, monitored the activity performed by patients, and dialogued with them if necessary. 86% of participants rated the TC training program positively. The pedometer was used excellently, being used by the patients for 90% of the study period without any negative events. After 12 weeks, only the TC group showed significant improvements in daily steps and movement intensity, while both groups showed significant changes in quality of life (SF-36). In conclusion, TC in patients with LTx appears to be safe, feasible, and well-accepted.

### Metabolic diseases

The third study included in this review was the only one that applied TC in elderly patients suffering from metabolic diseases. In detail, Storch and colleagues [32] applied TC in patients with type 2 diabetes mellitus (T2DM). Participants were recruited and randomized into two groups (i.e., the intervention group with the TC training program and the control group with usual care). The TC training program lasted 12 weeks and, through a tablet, a glucometer, and a pedometer, different measures relating to diet, PA, and stress management were recorded. In each phone-based meeting, the participant and the coach individually decided on the patient’s periodic goals to manage the disease. The number of daily steps was monitored continuously through the pedometer and automatically reported each day through Bluetooth. The results showed a greater reduction in glycated hemoglobin (HbA) values in the experimental group compared to the control group. In addition, the analysis conducted on PA demonstrated, although not significant, an increase in terms of average daily steps for the intervention group compared to the control group. However, this increase allowed a significant improvement in the management of the body mass index. It follows that a TC training program could be a new approach to manage disease progression in patients with T2DM.

## Discussion

This systematic review aimed to analyze the use of TC training programs in elderly people. Indeed, the average age of the world population is constantly growing and epidemiological studies showed that the number of people aged 60 or more exceeds 11% and is expected to increase reaching 22% in 2050 [33].

This aging process causes an increase in the development of age-related diseases and respiratory and metabolic diseases. One of the major respiratory diseases is COPD. This disease is characterized by bronchial obstruction with a progressive increase in dyspnea generating large physical limitations. For this reason, one of the main objectives to contrast this disease is to limit the sedentary lifestyle. This is demonstrated by the fact that physically active people with COPD have a lower risk of hospitalization and death than sedentary ones. Despite the demonstrated benefits of PA, these patients are still largely sedentary [34]. It follows that exercise professionals should counter the misconceptions related to the practice of PA or exercise in order to motivate individuals to train [35]. In this way, Cameron-Tucker and colleagues applied TC to these patients. Although their results demonstrate that the use of a diary with weekly walking plans and 1 h of weekly exercise does not induce benefits, TC can still be considered a useful method to increase daily PA time. The non-significant results found could depend on the reduced duration of the program, the small sample size, and the small number of tests carried out to evaluate physical efficiency. The 8 weeks of duration with 7 calls for single individual does not seem to be sufficient. Indeed, a systematic review of elderly patients with various chronic diseases demonstrated that 12 or more calls in 6–12 months are effective in increasing PA and improving eating behaviour [36]. Moreover, this study agrees with the scientific literature that 1 h of supervised exercise is insufficient to generate improvements in exercise capacity, supporting that at least two weekly sessions are necessary to improve exercise capacity in these patients [37, 38].

A further disease analyzed in this review refers to elderly with LTx, i.e., patients with end-stage lung disease who require surgery. In recent years, the disease survival rate has improved significantly and the International Society for Heart and Lung Transplantation Registry reports a 5-year survival rate of 59% [39]. It follows that one of the main objectives is to improve physical functions and the related quality of life in this population [40]. The study by Hume and colleagues demonstrated the effectiveness of TC in these patients. In detail, 86% of the participants evaluated this intervention positively stating that this allowed them to increase PA time. On the contrary one study in COPD patients, with the same intervention program, had a 59% satisfaction level [41]. 47.8% of participants with COPD rated goal progression as “high” or “much too high” compared to only 14% of participants with LTx. [41]. These results seem to suggest a greater ambition in choosing physical goals in LTx patients than in COPD patients. This condition could be due to a greater consideration of the role of PA [42] and the clear progress that the practice of PA generates in the symptoms of these patients [43].

The effectiveness of TC in elderly patients was also analyzed in subjects with T2DM. Diabetes is a metabolic disease characterized by chronic hyperglycemia with impairment of carbohydrate metabolism caused by partial or non-secretion of insulin. T2DM is the most common form of diabetes mellitus reaching 90/95% of all patients with diabetes [44]. It is also predicted that patients with this disease could become 439 million by 2030 [45]. The main objective for these patients is to prevent the onset of complications to maintain a high quality of life index [46]. In order to reach this goal, blood sugar levels need to be checked periodically and the American Diabetes Association (ADA) recommends HbA values < 7% [46]. TC program applied by Storch and colleagues allowed a reduction in HbA values from 7% to 6.6%, demonstrating the success of this training method in these patients. In detail, TC intervention has made it possible to increase daily activity with a consequent reduction in a sedentary lifestyle. A further benefit is the prevention of cardiovascular disease and all other complications that lead to death, especially in males with T2DM. It should be noted that high levels of physical fitness are associated with greater insulin sensitivity favouring better management and control of diabetes [47, 48].

Although 30% of elderly people suffer at least one fall per year [49], no literature supporting the application of TC as primary method for preventing falls in the elderly was identified. As reported by Lord and colleagues, one-third of older people experience at least one episode of falling each year [50]. The prevention of falls, due to the constant aging of the world population, is one of the main public health problems. Falls, after road accidents, are the main cause of death related to injuries among elderly subjects in the world [51], resulting in one of the main causes of death for people over 65 years of age [52]. In detail, high levels of PA reduce mortality and fall risk in the range of 30–50% [53–56].

## Conclusion

This systematic review highlighted that few studies have been conducted for evaluating the efficacy of TC training programs in elderly people. Despite this, the few studies included in this systematic review demonstrated that this training method seems to be safe, effective, and injury-free. In detail, the findings suggest that TC can be effective in subjects with metabolic diseases while partial results have been recorded in subjects with respiratory diseases.

Considering these results, further studies are needed to analyze the effectiveness of this intervention program. Moreover, future studies should investigate the efficacy of this training method also on different physical performance such as balance ability to evaluate its effects on falls prevention because TC program could increase adherence to the training, thanks to the possibility of overcoming barriers such as costs, time, travel, and facilities.

## Data Availability

Data are available upon reasonable request to the corresponding author.
